# Caesarean section on maternal request: a qualitative study of conflicts related to shared decision-making and person-centred care in Sweden

**DOI:** 10.1186/s12978-024-01831-z

**Published:** 2024-07-02

**Authors:** Mio Fredriksson, Inger K Holmström, Anna T Höglund, Emma Fleron, Magdalena Mattebo

**Affiliations:** 1 Department of Public Health and Caring Sciences, Health Services Research, Uppsala University, Box 564, Uppsala, 751 22 Sweden; 2 School of Health, Care and Social Work, Division of Caring Sciences and Health Care Pedagogics, Mälardalen University, Box 883, Västerås, 721 23 Sweden; 3 Department of Public Health and Caring Sciences, Centre for Research Ethics & Bioethics, Uppsala University, Box 564, Uppsala, 751 22 Sweden; 4 Akutmottagningen för gynekologi vid Akademiska sjukhuset, Akademiska Sjukhuset, Uppsala, 751 85 Sweden

**Keywords:** Caesarean section on maternal request (CSMR), Person-centred care, Obstetric care, Childbirth, Qualitative method, Patient autonomy

## Abstract

**Background:**

Today, person-centred care is seen as a cornerstone of health policy and practice, but accommodating individual patient preferences can be challenging, for example involving caesarean section on maternal request (CSMR). The aim of this study was to explore Swedish health professionals’ perspectives on CSMR and analyse them with regard to potential conflicts that may arise from person-centred care, specifically in relation to shared decision-making.

**Methods:**

A qualitative study using both inductive and deductive content analysis was conducted based on semi-structured interviews. It was based on a purposeful sampling of 12 health professionals: seven obstetricians, three midwives and two neonatologists working at different hospitals in southern and central Sweden. The interviews were recorded either in a telephone call or in a video conference call, and audio files were deleted after transcription.

**Results:**

In the interviews, twelve types of expressions (sub-categories) of five types of conflicts (categories) between shared decision-making and CSMR emerged. Most health professionals agreed in principle that women have the right to decide over their own body, but did not believe this included the right to choose surgery without medical indications (patient autonomy). The health professionals also expressed that they had to consider not only the woman’s current preferences and health but also her future health, which could be negatively impacted by a CSMR (treatment quality and patient safety). Furthermore, the health professionals did not consider costs in the individual decision, but thought CSMR might lead to crowding-out effects (avoiding treatments that harm others). Although the health professionals emphasised that every CSMR request was addressed individually, they referred to different strategies for avoiding arbitrariness (equality and non-discrimination). Lastly, they described that CSMR entailed a multifaceted decision being individual yet collective, and the use of birth contracts in order to increase a woman’s sense of security (an uncomplicated decision-making process).

**Conclusions:**

The complex landscape for handling CSMR in Sweden, arising from a restrictive approach centred on collective and standardised solutions alongside a simultaneous shift towards person-centred care and individual decision-making, was evident in the health professionals’ reasoning. Although most health professionals emphasised that the mode of delivery is ultimately a professional decision, they still strived towards shared decision-making through information and support. Given the different views on CSMR, it is of utmost importance for healthcare professionals and women to reach a consensus on how to address this issue and to discuss what patient autonomy and shared decision-making mean in this specific context.

**Supplementary Information:**

The online version contains supplementary material available at 10.1186/s12978-024-01831-z.

## Abstract (plain language summary)

Person-centered care is today a widespread approach, but accommodating individual patient preferences can be challenging, for example involving caesarean section on maternal request (CSMR). This study examines Swedish health professionals’ views on CSMR. Interviews with 12 health professionals reveal conflicts between CSMR and key aspects of person-centered care, in particular shared decision-making. While professionals acknowledge women’s autonomy, they question CSMR without medical need. Concerns include for example treatment quality and patient safety, and avoiding treatments that harm others. The Swedish context, balancing collective solutions with individualized care, complicates decision-making. Unlike countries with more private healthcare, where CSMR support might be higher, Swedish health professionals emphasize shared decision-making despite viewing the mode of delivery as primarily a professional decision. This study sheds light on the challenges in integrating CSMR into person-centered care frameworks.

**Supplementary Information:**

The online version contains supplementary material available at 10.1186/s12978-024-01831-z.

## Introduction

Today, person-centred care is seen as a cornerstone of health policy and practice and is considered a fundamental element of high-quality healthcare [1–4]. Central to person-centred care is a focus on the whole person [5] and a partnership between patients, their families and carers, and health professionals [2, 6]. Thus, the therapeutic relationship between the health professional and the person (including families and care partners) is at the centre, underpinned by values of respect for the person, individual right to self-determination, mutual respect and understanding [7]. Person-centred care in maternity services has the same foundations and objectives, emphasising that women’s values guide the decision-making before, during and after childbirth [8]. The key domains are dignity and respect, communication and autonomy, and supportive care [9].

However, person-centred care does not mean that individuals can receive exactly the care they want [10]. Rather, it means recognizing people's capabilities [11] and encouraging patients to clarify their own goals and work together with the health professional to identify the help they need to achieve these goals, which requires a good understanding of each other’s priorities [6]. In practice, treatment decisions also incorporate professional judgement, ethical considerations, system limitations etc. The last of these may be particularly apparent in health systems that are publicly funded and in which care is prioritised according to need. In the literature, ethical conflicts that may arise in the application of both person-centred and patient-centred care have been discussed in relation to holism, personal relationships and shared decision-making [12]. Paradoxes—i.e. simultaneous advantages and disadvantages from person-centred care—have also been found involving patient well-being, patient-provider interactions, work environment and costs [13].

An instance in which accommodating individual patient preferences can be particularly challenging is caesarean section on maternal request (CSMR), i.e. ‘elective delivery by caesarean section at the request of a woman with no identifiable medical or obstetric contraindications to an attempt at vaginal delivery’ [14]. This is a contentious procedure, with significant variations between countries and continents [15]. According to the WHO Statement on Caesarean Section Rates, maternal and neonatal mortality decrease up to 10–15% of births, while no such reductions are associated with a higher rate [16]. In contrast to areas such as Latin America and the Caribbean region, where rates of caesarean section (CS) have recently increased to about 40%, the Nordic countries have managed to keep CS rates low [17], including Sweden, which is well below the average in more developed countries (17.4% compared to 27.2%), Table 1 [18]. In this article, we explore the perspectives of Swedish health professionals on CSMR and analyse them with regard to potential conflicts that may arise from person-centred care, specifically in relation to shared decision-making. Shared decision-making can be seen as the middle ground between two dominant models of medical decision-making: paternalism and the patient’s informed choice [12], but what this entails may differ by healthcare setting [19]. It is a key component in both person-centred and patient-centred care [7, 20].

**Table 1:** Caesarean section (CS) rates by region and country^a^

**Region/subregion/country**	CS rate (%)	95% CI	Range (min–max, %)
**Africa (*n* = 44)**	9.2	5.2–13.2	1.4–51.8
Northern Africa (*n* = 5)	32.0	5.9–58.2	9.1–51.8
Sub-Saharan Africa (*n* = 39)	5.0	3.5–6.6	1.4–50.7
**Asia (*n* = 40)**	23.1	19.9–26.3	3.5–55.3
Central Asia (*n* = 5)	12.5	6.5–18.4	5.3–18
Eastern Asia (*n* = 5)	33.7	27.3–40.1	12.9–39.1
South-eastern Asia (*n* = 8)	15.9	9.6–22.3	3.5–32.7
Southern Asia (*n* = 7)	19.0	13.7–24.3	6.6–40
Western Asia (*n* = 15)	31.7	22.7–40.6	4.8–55.3
**Europe (*n* = 38)**	25.7	23.4–28.0	14.9–46.9
Eastern Europe (*n* = 10)	25.0	18.7–31.3	17.9–46.9
Northern Europe (*n* = 10)	25.3	21.5–29.1	15.9–32.6
Denmark	19.5		
Finland	16.4		
Iceland	18.3		
Norway	16.1		
Sweden	**17.4**	**Between 13–21% in the regions**^b^	
Southern Europe (*n* = 11)	30.1	27.5–32.7	21.2–34.1
Western Europe (*n* = 7)	24.2	18.3–30.2	14.9–32.7
**Americas (*n*** **= 25)**	39.3	34.6 – 44.0	5.4–58.1
Latin America and the Caribbean (*n* = 23)	42.8	37.6–48.0	5.4–58.1
Northern America (*n* = 2)	31.6	20.5–42.8	28.8–31.9
**Oceania (*n* = 7)**	21.4	6.6–36.2	3.0–34.6
Australia and New Zealand (*n* = 2)	33.5	1.9 – 65.1	27.9–34.6
Melanesia, Micronesia, and Polynesia (*n* = 5)	3.6	0.7–6.6	3.0–17.4
**World total (*n* = 154)**	21.1	18.8–23.3	1.4–58.1
More developed countries (*n* = 45)	27.2	25.2–29.2	14.9–55.3
Less developed countries (*n* = 70)	24.2	20.9–27.5	2.4–58.1
Least developed countries (*n* = 39)	8.2	5.2–11.2	1.4–32.7

^a^Comparative statistics for caesarean section on maternal request are difficult to find. The majority of the data presented in this table comes from Betran AP, Ye J, Moller A-B, et al. Trends and projections of caesarean section rates: global and regional estimates. BMJ Global Health 2021;6:e005671. doi:10.1136/ bmjgh-2021–005671. Data for the Nordic countries are added from Global Health Observatory data repository, last updated 2018–04-09 (Births by caesarean section: Data by country)

^b^[21]

Sweden is an intriguing case to study CSMR because Swedish health professionals find themselves in a complex landscape with conflicting policies, incentives and societal changes. On the one hand, current guidance on CSMR is restrictive [22] and the health system, funded through taxes, offers relatively few individual patient rights due to its redistributive nature, which is based on medical need [15, 16]. Swedish physicians also experience a low level of fear of legal consequences or being blamed in cases of adverse outcomes [23]. On the other hand, there is an overall wish to strengthen patients’ rights, and recent national policies promote person-centred care, a more individualised approach and increased patient choice [24]. There is also a loud social movement advocating for women’s right to choose CS as a mode of delivery, visible in both traditional and social media, influencing the national debate. Thus, there are forces that may simultaneously pull health professionals in different directions, leaving room for variation and uncertainty regarding CSMR practice.

Before describing the methodological approach, we summarise the literature on variations in attitudes towards CSMR and why women request elective CS, and describe the current regulations in Sweden. Further, shared decision-making is defined in the section describing the analytical framework.

### Non-medical and medical perspectives on CSMR

CS based on non-medical indications accounts for a large share of the global increase in CS [25, 26], but has no evident improvement in maternal and perinatal outcomes [15, 27]. When complications occur, CS is a life-saving intervention for women and newborns [25]. When performed based on non-medical indications its value is more difficult to assess, but a recent study indicates that the risks of short-term maternal complications are higher compared to vaginal delivery [28]. Generally, the frequencies of maternal mortality and maternal morbidity are higher after CS than after vaginal delivery. CS is associated with increased risks of uterine rupture, abnormal placentation, ectopic pregnancy, stillbirth and preterm birth. Evidence shows that children born by CS have different hormonal, physical, bacterial and medical exposures, and that these exposures can slightly change neonatal physiology. Short-term risks from CS for children include changed immune development and an increased likelihood of allergy and asthma [29].

The most common reasons women request CS are fear of labour pain, fear of birth, fear of urinary incontinence, fear of pelvic floor and vaginal trauma, and anxiety about infant injury/death [30]. But the doctor’s suggestion is also a factor [30, 31]. In general, women who request CS have higher levels of antepartum depression and anxiety [32], and women who wish to have a CS but deliver vaginally report higher levels of depression and PTSD after childbirth [32].

### Variations in attitudes towards CSMR

In general, midwives have a more restrictive attitude towards CSMR than obstetricians do [33]. Furthermore, professionals who work privately or have a private practice are more willing to perform CSMR [34], while female physicians are less likely [35]. Habiba et al. [36] conclude that differences in obstetricians’ attitudes towards CSMR are not founded on concrete medical evidence but rather stem from cultural factors and system differences in perinatal care organisation and legal liability.

In addition to the differences in attitudes and practices mentioned above, views on CSMR differ significantly between health professionals and pregnant women. Health professionals are less likely to be in favour of CSMR [37]. Although some variation exists, it has been found that obstetricians and midwives perceive that a major factor behind decisions to perform a planned CS is maternal request [38, 39]. According to Panda et al. [38], women’s requests are shaped by cultural beliefs, a perception of CS as a safe option for childbirth, and a lack of knowledge and awareness of risks. Romanis [40], however, contends that CSMR ‘is often inappropriately presented as unduly risky’ and calls for more discussion about why the benefits perceived by individual women are not recognised by clinicians.

### CS and CSMR in Sweden

The divide between health professionals and women requesting CS is clear in Sweden. A loud group hold that women have the right to CS (see e.g. Rätten att välja kejsarsnitt; Birth Rights Sweden (https://www.birthrightssweden.se/), while according to a recent report health professionals have differing views [21]. The women who hold that they have the right to CS believe that a CS implies a lower risk than vaginal delivery [21]. A 2006 survey shows that one third among the public agreed that women should be free to choose CS [41], but more recent information is lacking.

Traditionally, the Swedish maternity care system has had a culture of belief in ‘normal birth’ (a birth that starts naturally and does not involve medical or technological intervention). There is a belief among obstetricians and midwives that normal birth offers women and infants the best possible outcome [23, 42]. Midwife-led care using a team approach with the common goal of normal birth is a key element in maintaining a low CS rate in Sweden [23], and previous research has illustrated that when encountering women who request CS, midwives and obstetricians try to balance between resistance against a ‘risky project’ and respect [43]. Although Sweden has low CS rates, among the lowest in Europe [23], they increased from 10 to 17% from the early 1990s to 2015. Most planned CS in 2015 were performed on maternal request, an increase to 4.6% of all deliveries [44]. In a study from 2018 [23] it was indicated that fear of birth was not considered to be a major influencing factor in the decision-making process regarding CS in Sweden; nor were external influences such as the media or fear of legal consequences.

The law does not allow women to choose their mode of delivery [43], even if it stipulates that the patient’s autonomy and integrity is to be respected (SFS 2017:30 Chap. 4, 1§) and that healthcare shall, as far as possible, be formulated and carried out in consultation with the patient (SFS 2017:30 Chap. 5, 1§). Current guidance states that a woman must have a *sufficiently compelling reason* to have CSMR, which is to some extent open to health professionals’ individual interpretation [22]. It also specifies that a high degree of fear of birth can be a factor, as can acute psychiatric illness and being a victim of sexual abuse; but not age in itself, not previous CS, not previous birth injuries such as significant tearing and incontinence (unless they are persistent), usually not a previous stillborn or injured infant related to pregnancy or delivery, and not practical reasons such as planning. A woman’s request for a CS should be accommodated if her reasons are regarded sufficiently compelling and she maintains her request after receiving relevant information and being offered supportive discussions or other forms of care [22].

## Methods

### Design

A qualitative study using both inductive and deductive content analysis was conducted based on semi-structured interviews [45].

### Data collection

Interviews were held with 12 health professionals – seven obstetricians, three midwives and two neonatologists – working at different Swedish hospitals in southern and central Sweden. The purposeful sample was constructed to reflect the perspectives of different health professionals working at different hospitals on both the health and care of the pregnant woman and the child, with a majority of obstetricians included because they are the final decision-makers on CSMR. The interviews lasted between 25 and 60 minutes and were recorded and transcribed verbatim by authors and a professional transcriber. Audio files were deleted after transcription, in which only health professional status was the identifier. Ten interviews were conducted in March–April 2022 and the additional two in February–March 2023, between which times no changes took place in policy or regulations. The reason for adding two additional interviews was to achieve a higher saturation in the opinions expressed by obstetricians.

The interviews were conducted by authors EF and MM, without substantial differences in interview outcomes. The same interview guide was used for all interviews (Supplementary Material 1). Participants were contacted via email, informed about the study’s purpose and voluntary character, and asked whether they wanted to participate. They were informed that no names or hospitals wold be included when reporting the results, to maintain anonymity. The participants who signed consent to participate were included. This meant that we adhered to the Swedish Research Council’s four main requirements for protecting individuals: providing correct information, consent to participate, confidentiality and using the collected data only for the study purpose. Because the study does not collect or analyse sensitive personal data, according to Swedish Law (SFS 2003:460) no ethical permission was necessary.

### Analysis overview

As a first step, the interview material was categorised without using any predetermined categories or subcategories (inductively). MF and MM read all transcripts in detail and chose two transcripts that were then subjected to tentative categorisation by authors MF, IKH, AH and MM. These authors met and discussed the tentative categories and codes. MF and MM then performed a second round of coding and categorisation, and reviewed and revised the categories and subcategories. Lastly, MF and MM went back to all the transcripts and ensured that the codes in each category were coherent and could be clearly distinguished.

Thereafter, the categories were deductively analysed in relation to the potential conflicts as regards shared decision-making that may arise in the application of patient-centred care/person-centred care as defined by Hansson and Fröding [12]. Although person-centred care may have a more pronounced focus on the whole person [5], Hansson and Fröding view patient-centred care and person-centred care as concepts with closely related meanings. Other scholars [20] who have compared the two concepts also conclude that although there are some significant differences between patient- and person-centred care, for example relating to the goals (a meaningful life and a functional life, respectively), there are also many similarities. Both involve empathy, respect, engagement, relationship, communication, holistic focus, individualized focus, coordinated care as well as *shared decision-making*.

### Shared decision-making: analytical framework

Shared decision-making can be seen as the middle ground between two dominant models of medical decision-making: paternalism and the patient’s informed choice [12]. Paternalism implies that physicians (or other health professionals) know better than the patient what is in their best interest and should thus be the ones who ultimately make the decisions. This has been challenged by the concept of the patient’s informed choice, in which the physician’s role is to offer adequate diagnostics and inform and advise the patient, but leave the final decision to the patient. Shared decision-making, as a middle ground, emphasises that a decision must be an agreement between patient and health professional after they have both shared their information and treatment preferences. However, it does not imply that both parties must be convinced that a particular treatment is the best possible option for the patient [12]. Montori et al. suggest that implementing shared decision-making in practice involves fostering conversation, purposefully selecting and adapting the process (matching preferences, reconciling conflicts, problem solving and meaning making), support, and evaluation and learning [46].

Shared decision-making is generally seen as component of person-centred care as well as patient-centred care [7, 20], although some have argued that the patient’s informed choice seems to be better aligned with ensuring that patient preferences and needs guide all clinical decisions. The potential conflicts identified by Hansson and Fröding relate to shared decision-making versus (i) patient autonomy, (ii) treatment quality and patient safety, (iii) avoiding treatments that harm others, and (iv) equality and non-discrimination. We analysed our inductively developed categories in relation to these conflicts, and added an additional potential conflict that was present in the data: v) shared decision-making versus an uncomplicated decision-making process.

Trustworthiness (credibility, transferability, dependability, and confirmability) is discussed at the end of the discussion section.

## Results

Examples of all four potential conflicts emerged in the interviews, with the most categories related to the conflict between shared decision-making and patient autonomy. We also found an additional category: shared decision-making versus an uncomplicated decision-making process, Table 2. In total, we found five potential conflicts (categories) and twelve expressions of these conflicts (subcategories).

**Table 2:** Potential conflicts relating to shared decision-making and their expression in Swedish health professionals’ views on CSMR

Potential conflicts	Expression of potential conflicts in the interviews
Patient autonomy	• The right to decide over one’s own body
	• Fear of birth is not a question of autonomy
	• Healthcare services on demand
	• Challenges to professional autonomy
	• Preferences shaped by media reports and social media platforms
Treatment quality and patient safety	• The woman’s future health
	• Previous birth experiences matter
Avoiding treatments that harm others	• Costs for society/the health system
	• The child’s perspective is secondary
Equality and non-discrimination	• Strategies for avoiding arbitrariness
An uncomplicated decision-making process	• A multifaceted decision being individual yet collective
	• Birth contracts to increase sense of security

The table draws on Hansson and Fröding’s [12] categorisation of the potential conflicts that may arise in the application of patient-centred/person-centred care

### Patient autonomy

The health professionals’ reasoning about CSMR can be attributed to several aspects of patient autonomy. There was a moral aspect involving the right to decide over one’s own body, a denial of fear of birth as a matter of patient autonomy, reference to the law in relation to demand, a perception of a challenge to their professional autonomy, and an observation that preferences for CSMR were affected by reports in the media and on social media platforms about crises and shortcomings in maternity and delivery care.

#### The right to decide over one’s own body

None of the health professionals questioned the notion that women should have the right to decide over their own body, but the extent to which they felt this right includes CSMR varied. Some believed that women should have the right to choose their mode of delivery, provided they are fully informed about current and future risks. However, most of the professionals believed that women should not have the unrestricted right to choose CS, in the same way as people who use the healthcare services should not have the right to choose other operations without medical reasons. One reason cited for this was the potential of adverse medical consequences for the individual (patient safety), and another was that it could have adverse consequences for the system (harm others). Another type of reason was based on the belief in normal birth, with one of the midwives arguing that when you have made the choice to become pregnant, you must also accept the consequences. She said that, if the health services deny a CS, ‘*You cannot say we’re the ones who are committing an assault”* (I4). Nevertheless, it was emphasised that women should, of course, receive the support they need both before and during delivery, such as therapy and adequate pain relief.

However, the topic proved challenging for the professionals, who regarded it to some extent as a theoretical or philosophical question that was difficult to handle in practice. As one of the obstetricians said, *‘I think it’s really hard. I don’t have any definite answers, be it that you always have the right to have a CS or that we always have the right to deny’* (I8). Another professional believed that this is an issue that will never be resolved but will rather need to be continuously addressed.

#### Fear of birth is not a question of autonomy

The health professionals expressed that the vast majority of women requesting CS suffer from a fear of birth, which they did not consider to be a discussion about autonomy but rather as often being related to mental health issues. Reasons mentioned for CSMR requests included experiences of sexual assault, fear of extensive ruptures in the vagina, mental health issues and PTSD. Maternal mental health was mentioned often, and some of the health professionals believed this reflected a general increase in mental health issues in society. One midwife said that this had led to a behaviour of avoiding vaginal delivery instead of preparing for it, and that the societal development had led some women to think *‘that they could get help with everything’* (I4). Another midwife described these women as belonging to a ‘vulnerable group’.

It was a commonly held opinion that the desire for CS often stemmed from an underlying issue that could be addressed and overcome. Regardless of the reason, however, it was emphasised that it is important to understand why a pregnant woman expresses a fear of birth, especially if this is her first time being pregnant. The right to have individual help and support as early in the pregnancy as possible was described as important, and the midwives saw it as their role to provide this help.

#### Healthcare services on demand

According to the health professionals, a small but significant minority claimed the right to choose their mode of delivery. In some cases, this choice was influenced by being accustomed to paying for the healthcare treatments they desired. It was expressed that women’s backgrounds influenced differences in their beliefs regarding the right to make decisions about their own care:

*Then there’s a small minority, and these are people who come from countries where you can actually choose [to have] a caesarean section, and most of the time you have to pay for it yourself, but who come and say ‘I want a caesarean section, please,’ often without even specifying a reason for it…* (I12)

Pregnant women who came to the maternity services with the opinion that they should have the right to choose CS were considered most difficult to have a conversation with. In such cases, the discussion was not based on medical or psychological perspectives but rather values and norms. According to the health professionals, this led to difficulties, as pregnant women in Sweden do not have the right to choose their mode of delivery. It was described that there were Facebook groups, with a confrontational approach, supporting these women.

#### Challenges to professional autonomy

During the interviews, it became evident that the professionals saw CSMR as a challenge to their professional autonomy and individual decision-making. In general, they described that medical decision-making should involve both the professional and the patient, but they emphasised that the ultimate responsibility for making the decision rests with the professional. An obstetrician expressed the following:

*Once a year there’ll be a woman who says ‘I’m a woman, I’m pregnant, it’s my child and I have the right to make decisions about my body.’ And if I were a private person I could maybe nod and say ‘of course,’ but as an obstetrician I cannot; instead I have to say ‘according to rules and regulations, I’m ‘unfortunately’ the one who has to make the assessment and decision.’* (I10)

Although the professionals explained that their decisions had to align with current regulations and policies, this was not regarded as an easy decision. Several of them found it challenging to insist on vaginal delivery if a woman truly did not want to go through with it, due to a fear of what would happen if the delivery was difficult or if the woman suffered from psychological ill health because of it. Furthermore, there was some variation among the professionals regarding whether they viewed pregnancy and delivery as a unique condition or made comparisons with other medical treatments or surgical procedures. Several exemplified their perspective by pointing out that professionals make decisions about knee operations, throat surgery and so on, saying it should be no different when determining the need for CS. As one obstetrician put it:

*There are no other situations where you can choose whether or not to undergo surgery without a medical reason, at least not in publicly funded healthcare, in contrast to private healthcare or aesthetic surgery.* (I7)

However, others tended to see pregnancy and delivery as a distinct condition that required a different approach. For instance, one obstetrician argued that because women bear the burden of reproduction it should come with privileges, such as the right to terminate a pregnancy and the right to choose the mode of delivery.

#### Preferences shaped by media reports and social media platforms

Most of the professionals mentioned that the CSMR rate and preferences for CSMR were shaped by how women perceived the functioning of maternity and delivery care. Many women, they believed, felt they could not trust the healthcare services. The professionals also noted that media reports about crises in maternity and delivery care, the risk of severe birth injuries, and similar factors, influenced women’s preferences regarding opting for CS instead of vaginal delivery. One example they mentioned was the fear generated by media reports that there might be insufficient staff available during delivery, leading to poor quality of care. Another example involved reports that women could be denied access to their chosen hospital at the time of delivery due to overcrowding and hence be forced to go somewhere else to give birth.

Some of the professionals mentioned that women’s fears were to some extent justified, but resulted in too low a level of support during delivery rather than low patient safety. Having a guaranteed time and place for delivery increased women’s sense of security and control in these situations. According to the health professionals, this sense of security and control was associated with the request for CS. One midwife expressed it this way:

*…they have a full surgery team that gives them full attention and a midwife who’s present and takes care of the child, and who doesn’t have to run between deliveries. I wish we could put an equal amount of resources into vaginal deliveries.* (I2)

In contrast, one of the obstetricians expressed the reality:

*…it’s very hard today to, in good conscience, look a woman in the eye and tell her that she’ll have the support and presence she needs during the delivery.* (I7)

In alignment with this, another obstetrician pointed out that maternity and delivery care services had previously failed to treat severe tear injuries adequately and that, in general, maternity and delivery care had been deprioritised in research as well. The obstetrician acknowledged that the healthcare services had not previously given enough attention to postnatal problems and that women had the right to receive appropriate help to ensure a well-functioning body, which could reduce the desire for elective CS.

The health professionals also mentioned groups on social media platforms advocating for the right to choose CS, often as a response to concerns about poor maternity care and a high risk of injuries for women (and sometimes for the child). Some of the professionals tried to stay up to date on what aspects of CSMR were discussed in social media groups. One of the midwives, mentioning that she had been exposed on social media and perceived the tone of debate as heated and negative, said:

*In order to endure, one must feel secure in what one does, to be able to resist in some way, to believe in one’s cause, and constantly go back to that.* (I4)

### Treatment quality and patient safety

Treatment quality and patient safety were embedded in much of the health professionals’ reasoning. However, there were instances in which their opinions differed from those of the women or there could be a conflict between the short and the long term. They had to protect the woman’s future health and consider her previous birth experiences.

#### The woman’s future health

Consideration for the woman’s future health was an important aspect for the health professionals. They explained that they could not focus solely on the woman’s immediate desires and reasons, but also had to consider her future health and potential pregnancies. Many of them discussed how a CS could impact future CS procedures and other abdominal surgeries, and said they made a point of discussing this with the pregnant woman. Sometimes the woman experienced this as persuasion or intimidation. The health professionals also mentioned their preference for women to give birth vaginally in order to improve their long-term health and obstetric history. An obstetrician exemplified how they explained to a woman the potential future risks associated with CS, saying:

*…having had abdominal surgery, if you get cancer later on and maybe have a lot of abdominal adhesion making it difficult to operate, or in future pregnancies, if the placenta grows into scar tissue and you’re forced to undergo an acute hysterectomy, to remove the uterus with a high risk of bleeding.* (I8)

#### Previous birth experiences matter

The health professionals expressed that the difficulty of meeting the needs of pregnant women varied depending on their previous birth experiences. A woman who is pregnant for the first time should primarily be guided to a vaginal delivery, while providing a secure and safe plan. The care for women requesting CS included supportive conversations with midwives and/or psychologists. However, for multiparous women with prior experiences of difficult vaginal delivery or emergency CS, it was more likely that a CS would be granted early on. One obstetrician discussed this:

*For a young first-time mother, we work very hard to try to make her feel secure with a vaginal delivery because she may have many pregnancies and deliveries ahead of her with the significant risks associated with repeated CS. In contrast, if I have a multiparous woman who has a very traumatic childbirth experience behind her and she’s 40 years old, perhaps it’s not worth putting all our effort into that woman [working towards a vaginal delivery], especially if she’s previously had a CS due to lack of labour progress.* (I7)

Desire was expressed for additional resources in order to be able to support women with previous traumatic birth experiences. Sometimes vaginal delivery was considered possible from a medical standpoint, regardless of previous birth experiences, but as resources for psychosocial support were often lacking a CS could ultimately be performed instead.

### Avoiding treatments that harm others

The health professionals mentioned two aspects that involved reasoning about avoiding treatments that harm others. They considered costs on a broader level and wanted to avoid crowding-out effects, but saw the child’s perspective as secondary to that of the pregnant woman.

#### Costs for society/the health system

None of the interviewed health professionals believed that costs played a role in individual decisions about CSMR, although they acknowledged that cost considerations were relevant at the societal level. One of the midwives said:

*No, we only think about the woman. We don’t take into account potential health-economic effects, even if I believe there are such effects.* (I4)

Similarly, an obstetrician said:

*It’s about the individual in front of me, the best way for her to become a mother, for her and her family.* (I9)

However, many professionals incorporated a broader societal perspective into their reasoning, arguing that unrestricted CS might have negative effects on other patients and patient groups by causing crowding-out effects. They contended that it could lead to an increase in CS rates and cause other conditions that would demand healthcare resources.

#### The child’s perspective is secondary

When the child’s perspective was considered, it was only mentioned as a reason for CS in cases of illness or identified risks. If no medical conditions were present, there were no grounds for considering the child’s perspective when deciding on CS. One neonatologist expressed this as follows:

*It’s something between the maternity care services and the pregnant mother; there’s no basis, from the newborn’s perspective, for imposing an opinion on whether a planned CS is warranted or not*. (I6)

In comparison to a vaginal birth, the health professionals mentioned that children born via CS might experience mild adjustment problems. However, they found it challenging to establish a direct causal link between CS deliveries and potential conditions or illnesses in infants, and considered CS more detrimental to the woman.

### Equality and non-discrimination

Although the health professionals emphasised that every CSMR request was addressed individually, they referred to different strategies for avoiding arbitrariness.

#### Strategies for avoiding arbitrariness

In some clinics, in order to prevent arbitrariness obstetricians never made decisions themselves, for example because some women can be more persuasive or well read than others. This was also part of a strategy for preventing obstetricians and midwives from developing a reputation for being more accommodating, which could spread on social media and might make it easier for women to advocate for a positive decision regarding CS. A midwife who had worked in several regions said that among the obstetricians she had worked with, some had been more ‘generous’ while others had ‘stood their ground’.

Other reasons mentioned for why a team-decision approach was practised involved possible co-morbidities. This meant that different competences contributed different expertise, and were considered important in reaching the right decision for the women. Challenges involved in making assessments regarding the necessity of CS included, for example, a lack of training in conducting such assessments. An obstetrician related the following:

*I don’t have a formal psychiatric education, and the ability to assess people’s mental characteristics is probably more on an amateur basis. Unfortunately, I think this applies to quite a few obstetric gynaecologists. Our knowledge in psychology and psychiatry leaves something to be desired…* (I11)

### Uncomplicated decision-making processes

An additional potential conflict that could be detected in the interviews with the health professionals was that between shared decision-making and an uncomplicated decision-making process. They described that CSMR entailed a multifaceted decision being individual yet collective, and the use of contracts in order to increase a woman’s sense of security.

#### A multifaceted decision being individual yet collective

One important aspect throughout the interview material was the approach of individual assessment within a collective environment. All of the health professionals strongly emphasised that all decisions were individual and based on the specific woman’s situation, although the professionals themselves felt that the vaginal mode of delivery was preferable. If the assessment indicated that the pregnant woman wanting a CSMR could manage a vaginal delivery, the process focused on enhancing coping strategies for a vaginal delivery. However, if the assessment led to doubts, the woman was granted a CS. The significance of teamwork before decisions were made was stressed, and many mentioned that they discussed decisions within specific CS teams. One of the obstetricians described how the team was consulted:

*… for those who I believe have the ability to handle it, I try to guide them towards a vaginal delivery. For those who I assess do not have that ability, I then consult our team and suggest that we opt for a CS for this individual.* (I11)

Many perspectives and methods for reaching a decision were described, for example regarding the timeline. Some clinics practised a fast decision process, while others preferred a longer period between initial request and decision. Delaying the decision made it possible to provide pregnant women with support, offer them information about complications related to CS, and attempt to guide them towards a vaginal delivery, if feasible. It was also considered important to impart knowledge and information to support an informed choice regarding CS. A midwife emphasised the importance of understanding the risks:

*Yes, but one should also make a very active choice, and one must understand what one is risking (…) it must be that you’ve understood what kind of risks we’re subjecting you and the child to by performing a CS.* (I3)

#### Birth contracts to increase sense of security

In some cases, ‘birth contracts’ were used as a means of establishing a mutual understanding between the women and the staff. In these contracts, the pregnant women agrees to first attempt a vaginal delivery, with the possibility to convert to a CS under certain conditions. It was stressed that these contracts were to be followed provided that the medical conditions allowed for this. It was also deemed important to communicate realistic expectations as to the contract’s validity, as failing to do so could result in disappointment and, potentially, negative birth experiences:

*I think most Swedish clinics use them. But it’s like this: all these plans and agreements are valid under the conditions that apply when the decision was made. I usually make it very clear that this isn’t a game, and if medical circumstances require deviating from the plan to ensure the health of the child or the mother, we’ll do it safely and without regard for what we’ve agreed on. Because life comes before health, and health comes before anxiety and fear… But generally speaking, we try to develop plans that are realistic and achievable…* (I11)

In summary, contracts were employed to enhance both trust and a sense of security among the women. The contracts also informed all staff at the delivery wards, regardless of their profession, about the individual’s plans and agreements. This too was deemed to increase trust and a sense of security in the women. For all involved parties, the ultimate objective was to guide each woman towards a positive birth experience, irrespective of whether it involved CS or vaginal delivery.

## Discussion

In some ways, the findings discussed in this article correspond to previous studies from Sweden [23, 43], illustrating a culture of belief in normal birth, a disapproval of fear of birth as a relevant factor for elective CS, and a focus on supporting women in having a safe delivery, with midwives playing a crucial role. The findings also support the notion that obstetricians make the final decision regarding the mode of delivery, often through a team approach, which our study suggests is an approach that aims to prevent arbitrariness as well as protect individual health professionals. However, in contrast to a previous study [23], we also find that media portrayal of the maternity and delivery care services, and discussions on social media platforms, influence CSMR practice as they contribute to shaping a preference for it among Swedish women. Our analysis also illustrates that the health professionals’ perspectives on CSMR reveal potential conflicts that may arise from person-centred care, particularly shared decision-making, even if not explicitly asked to identify such conflicts. Shared decision-making is usually defined as a collaborative approach, by which patients together with clinicians are encouraged to think about different treatment and care options, benefits and harms with these, to communicate their preferences and select the best course of action. Below, we summarize the conflicts and relate to the four ways a patient’s problematic situation can be addressed together by patients and clinicians in shared decision-making as mentioned by Montori et al. [46]: 1) matching preferences, 2) reconciling conflicts, 3) problem solving and 4) meaning making.

Patient autonomy is one of the leading ethical principles of medical practice today [47], which Romanis ([48], p. 255) argues should not be “diminished by pregnancy”. However, the conflict involving shared decision-making versus (i) *patient autonomy* was a stumbling block for many health professionals in our study, not least because it challenged their professional autonomy and thus their professional preferences. Although most health professionals agreed in principle that women should have the right to decide over their own body, they did not believe this included the right to choose surgery without medical indications. This contrasts with previous research that found the vast majority of obstetricians believed women should be able to choose CSMR [49], illustrating the need for a public debate to explore how patient autonomy should be practiced in this area within healthcare.

Most of the health professionals dismissed CS due to a fear of birth as a question of patient autonomy, but rather saw this as a preference affected by media portrayal and social media groups. However, women using their autonomy and requesting CS could be seen as a way to handle their lack of trust in the health services (in part created by the media’s focus on shortcomings and deficiencies, resulting in things like severe tear injuries), as they regard this as safer than the vaginal mode of delivery [21, 38].

The conflict between shared decision-making and (ii) *treatment quality and patient safety* was also embedded in the interviews. It is clear that the interviewed health professionals regarded vaginal delivery as medically beneficial (in the absence of medical indications), and saw potentially harmful effects from CS, thus impacting on the principle of beneficence/non-maleficence, i.e. to minimize harms and maximise benefits [48]. The obstetricians – who are responsible for not harming the patient and for the effects of medical interventions [12] – expressed concerns that CSMR would lead to adverse medical consequences in the short term, but also expressed that they have to consider the woman’s future health as well, which could be negatively impacted by unnecessary CS. However, treatment quality and patient safety might mean different things to the health professionals and the women wanting a CS, in line with Romanis’s [40] suggestion that the benefits perceived by women requesting elective CS are not always recognised by the health professionals. In the public debate, women advocating for the right to choose CS argue, for example, that the current evidence does not include psychological effects from, for instance, being denied autonomy or living with severe delivery injuries [21, 37]. In terms of shared decision-making, when a woman requests a CSMR in Sweden, it is not a situation with a number of options potentially *matching the patient’s preferences*, and where the patient and clinician can deliberate until the best match is identified, but rather a decision where the patient and the clinicians often have different preferences. However, to a higher extent than suggested by national guidance (which disregards, e.g., previous birth injuries), the health professionals seemed to take into consideration previous birth experiences.

The health professionals’ reasoning also involved examples of the conflict between shared decision-making and (iii) *avoiding treatments that harm others*. This was expressed in terms of a conflict between individual and collective/societal perspectives (the justice principle [48]), but not as one between mother and child – the child’s perspective being secondary when there were no medical indications. Furthermore, the health professionals argued that individual decisions were never influenced by cost considerations. They did consider costs in a broader sense, however, and expressed that CS procedures are more expensive and can lead to crowding-out effects. For example, other surgeries may need to wait due to the demand for CS, and CS can result in other conditions that require healthcare resources, such as post-operative infection. This is closely tied to the conflict involving shared decision-making versus (iv) *equality and non-discrimination*. It is commonly believed that equality and non-discrimination imply that resources should be allocated based on need rather than demand, which poses a challenge when it comes to CSMR. A team-decision approach was used to avoid arbitrariness in CSMR decisions, which may reduce inequalities in how CSMR requests are handled by various health professionals. One can argue that shared decision-making holds a potential risk for conflicts and disagreements, as well as difficulties in reaching a consensus between health professionals and women requesting a CS. This could, on the other hand, increase inequality. In contrast, a review concluded that even though women want to choose the mode of delivery, with the safety of their babies as the priority, they also trusted the advice of their maternity care providers and considered it the responsibility of their obstetricians to make the decision [50].

In the interviews we also detected a conflict between shared decision-making and (v) *an uncomplicated decision-making process*. It was emphasised that the CSMR decision was multifaceted, being both individual and collective. A team approach was common, and a prolonged decision process was often employed to allow time for informing and supporting the woman, with a preference for vaginal delivery. In terms of shared decision-making, this can be seen as an attempt to *reconciling conflicts* [46], i.e. helping the patient articulate her reasons for her treatment preferences (in this case CS), and reconciling these reasons with treatment possibilities, mainly through information. However, importantly, for women requesting CS without medical reasons, their perspective on what was best for them was often discussed without their presence, potentially reducing the conditions for meaning-making [46], i.e. why pursuing a particular approach. Women requesting CS should be presented with possibilities to ask questions in order to avoid misunderstandings as to why different decisions are made [51]. Shared decision-making implies that both parties agree to a certain treatment or course of action, although both parties may not necessarily be convinced that it is the best solution. This may require *problem solving* [46]. To achieve agreement, birth contracts (sometimes referred to as CS contracts) appeared to be a common solution. In these contracts, the pregnant woman agrees to first attempt a vaginal delivery, with the possibility to convert to a CS under certain conditions. This does not imply that the pregnant woman believes this is the best option for giving birth but rather that she can consent to this course of action. In this case, problem solving as well as reconciling conflict and meaning making were also achieved through comprehensive efforts to support and prepare women for a vaginal birth and through counselling prior to CSMR decisions.

To summarise, the complex landscape for handling CSMR in Sweden, arising from a restrictive approach centred on collective and standardised solutions, alongside a simultaneous shift towards person-centred care and individual decision-making, was evident in the health professionals’ reasoning. We would probably not find this complex landscape in countries with more private healthcare – where higher percentages of stakeholders, particularly obstetricians, support CSMR – or in countries with more individualised patient rights and private funding [50]. However, research from the UK has shown that although recommendations are more permissive than in Sweden, suggesting that a competent woman’s request should be respected, women are routinely denied elective CS [48]. In the interviews from Sweden we found instances of a traditional paternalistic model, whereby physicians (and other health professionals) are presumed to know better than the patient what is in her best interest. In this case, they favoured vaginal delivery over CS. In contrast, we found no support for a fully informed choice model in which the patient is left to make the final decision after the physician has provided information and advice. Nonetheless, the interviewed health professionals regarded it as essential to inform patients about risks. While the instances of paternalism and a lack of fully informed choice suggest that patient participation and empowerment in relation to CSMR are rather low, the interviews indicate that many health professionals strive towards shared decision-making in which the decision has to be an agreement between the patient, in this case the pregnant woman, and the health professional. The use of contracts was an attempt to achieve this, even if it does not uphold the woman’s preference. The professional relationship between patient and health professional should be based on a moral level of shared autonomous rights as well as a responsibility to respect each other. Even if women do not have the right to independently demand the kind of delivery they will have, a shared decision-making process is desired [21].

Lastly, this study has some limitations. It is relatively small-scale, and due to the specific conditions pertaining to Swedish healthcare it may be difficult to generalise the results to other health systems. The results are transferable within the Swedish setting due to the mix of health professionals working at different hospitals, but another study should also include psychologists and psychiatrists who sometimes are consulted in the CSMR decision-making process. However, since Sweden is a country with a CS rate well below the average in more developed countries, it should be seen as an example of a country with a restrictive approach to CSMR, together with the other Nordic countries. Further, regarding trustworthiness, credibility was achieved by using the multi-professional research group’s different perspectives to reflect on personal biases or preconceptions. Dependability was sought by documenting the research process and confirmability by and presenting quotations to support the analysis, continuously discussing the analysis and interpretations in the author group and by presenting them to other researchers to minimizing researcher bias.

## Conclusions

The complex landscape for handling CSMR in Sweden, arising from a restrictive approach centred on collective and standardised solutions, alongside a simultaneous shift towards person-centred care and individual decision-making, was evident in the reasoning of the health professionals. Their perspectives on CSMR illustrated a number of conflicts that arise from person-centred care. Most evident were those related to patient autonomy, treatment quality and patient safety, as well as avoiding treatments that harm others. CSMR was perceived as an unsolvable dilemma and as a balance between the woman’s current and future preferences and health, and brought about a fear of crowding-out effects. Although most of the health professionals emphasised that it is ultimately a professional decision to make, they still strived towards shared decision-making through information and support. Given the different views on CSMR, it is of utmost importance for both health professionals and women to reach a national consensus on how to address this issue. The goal should never be to dissuade women from choosing a CS but, more importantly, to encourage them to make informed decisions with input from various specialists, regardless of where the woman lives. Legal requirements mandate the provision of equal care, which is currently not met due to the different approaches in each healthcare region in Sweden. Therefore, obstetricians, midwives, and mental health specialists should be part of the decision-making team. Simultaneously, we need to discuss what patient autonomy and shared decision-making mean in this specific context. Ultimately, the goal is a healthy mother with an overall positive birth experience and a healthy child. To achieve this, we need clear and unequivocal guidelines that are not influenced by personal views, ensuring that shared decision-making is a natural and integral part of respecting patient autonomy.

### Supplementary Information

Supplementary Material 1.

## Data Availability

The datasets generated and/or analysed during the current study are not publicly available due to confidentiality, but are available from the corresponding author on reasonable request.
