# Supplementary material for: Caesarean section on maternal request: a qualitative study of conflicts related to shared decision-making and person-centred care in Sweden
Source: Reprod Health. 2024 Jul 2;21:97. doi: 10.1186/s12978-024-01831-z (PMC11221017; doi:10.1186/s12978-024-01831-z)
Supplement: Supplementary file 1 — Supplementary Material 1. [file 12978_2024_1831_MOESM1_ESM.docx]

**Additional file 1: Interview guide**

**Caesarean section on maternal request: conflicts related to shared decision-making and person-centred care**

- What is your professional role in maternity- and delivery care?
- What is your role in deciding whether or not a woman who wants a caesarean section without medical indications is approved one?
- Does your workplace have any guidelines or policies on how decisions regarding caesarean section on maternal request should be made?
- Who is part of the team when deciding on a caesarean section on maternal request?
- How long does the process take to get an approval to undergo caesarean section without medical indications?
- Can you describe as detailed a picture as possible of a woman being approved for a caesarean section without medical indications?
- Can you describe as detailed a picture as possible of a woman where one cannot accommodate to perform a caesarean section on maternal request?
- How do you/your team weigh all the indications together and is there something that is more important then? (only obstetricians)
- From the child's perspective, how do you see the increased trend of caesarean section on maternal request? (only neonatologists)
- How do you think the current crisis in maternity care affects women's desire for caesarean delivery?
- How often do you come across requests for caesarean section without medical indications? Is it more frequent now compared to before? (only obstetricians and midwives)
- How often do you treat babies requiring neonatal care after caesarean section and caesarean section on maternal request? Is it more frequent now compared to before? (only neonatologists)
- What considerations do you/the team have to make during the assessment? What about, for example, the child, health economics or the capacity of the healthcare system?
- Some women state that they have a right to a caesarean section, what do you think of that statement?
